# Remdesivir for Patients Hospitalized with COVID-19 Severe Pneumonia: A National Cohort Study (Remdeco-19)

**DOI:** 10.3390/jcm11216545

**Published:** 2022-11-04

**Authors:** Jeremie Zerbit, Marion Detroit, Sylvie Chevret, Frederic Pene, Charles-Edouard Luyt, Jade Ghosn, Frederic Eyvrard, Guillaume Martin-Blondel, Benjamine Sarton, Raphael Clere-Jehl, Pierre Moine, Amelie Cransac, Pascal Andreu, Marie Labruyère, Laetitia Albertini, Jean-François Huon, Pauline Roge, Lise Bernard, Magali Farines-Raffoul, Maxime Villiet, Arnaud Venet, Louis Marie Dumont, Jean-Daniel Kaiser, Claire Chapuis, François Goehringer, François Barbier, Stephane Desjardins, Younes Benzidi, Nora Abbas, Corinne Guerin, Rui Batista, Jean-François Llitjos, Marie Kroemer

**Affiliations:** 1 Department of Pharmacy, Hospital at Home, University Hospitals of Paris, 75014 Paris, France; 2 Department of Pharmacy, University Hospital of Besançon, 25056 Besançon, France; 3 Department of Biostatistics, Saint-Louis Hospital, AP-HP, Universite Paris Diderot, INSERM S717, 75010 Paris, France; 4 Institut Cochin, Université de Paris, INSERM U1016, CNRS UMR 8104, 75014 Paris, France; 5 Service de Médecine Intensive et Réanimation, Hôpital Cochin, AP-HP, 75014 Paris, France; 6 Médecine Intensive Réanimation, Institut de Cardiologie, Hôpital Pitié-Salpêtrière, AP-HP, 75013 Paris, France; 7 INSERM, UMRS_1166-ICA, Sorbonne Universités, 75005 Paris, France; 8 Infectious Diseases Department, Bichat-Claude Bernard University Hospital, AP-HP, 75018 Paris, France; 9 Pharmacy Department, Toulouse University Hospital, 31300 Toulouse, France; 10 Department of Infectious and Tropical Diseases, Toulouse University Hospital, 31300 Toulouse, France; 11 Inserm U1043—CNRS UMR 5282, Toulouse-Purpan Pathophysiology Center, 31300 Toulouse, France; 12 Critical Care Unit, University Teaching Hospital of Purpan, Place du Dr Baylac, 31300 Toulouse, France; 13 Toulouse NeuroImaging Center, Toulouse University, Inserm, UPS, 31300 Toulouse, France; 14 Service de Médecine Intensive—Réanimation, Hôpital de Hautepierre, Hôpitaux Universitaire de Strasbourg, 67091 Strasbourg, France; 15 Intensive Care Unit, Raymond Poincaré Hospital, AP-HP, 92033 Garches, France; 16 Université Paris-Saclay, UVSQ, INSERM, Infection et Inflammation, 78180 Montigny le Bretonneux, France; 17 Department of Pharmacy, Dijon University Hospital, 21231 Dijon, France; 18 Department of Intensive Care, Dijon Bourgogne University Hospital, 21231 Dijon, France; 19 Pharmacy Department, Montfermeil Hospital, 93047 Montfermeil, France; 20 Clinical Pharmacy Unit, CHU de Nantes, 44000 Nantes, France; 21 Pharmacie, CHRU Brest, Hôpital de La Cavale Blanche, 29200 Brest, France; 22 Département de Pharmacie, CHU Clermont-Ferrand, 63000 Clermont-Ferrand, France; 23 Centre de Recherche Clinique, Centre Hospitalier Annecy Genevois, 74000 Annecy, France; 24 Département de Pharmacie, Centre Hospitalier Universitaire de Montpellier, 34000 Montpellier, France; 25 Department of Pharmacy, Pellegrin Hospital, 33000 Bordeaux, France; 26 Medical Intensive Care Unit, Louis-Mourier Hospital, AP-HP, 92025 Colombes, France; 27 Pharmacy Department, Hôpitaux Civils de Colmar, 68026 Colmar, France; 28 Clinical Research Unit, Hôpitaux Civils de Colmar, 68026 Colmar, France; 29 Unités Pharmacie Clinique et Médecine Intensive-Réanimation, Centre Hospitalier Universitaire de Grenoble Alpes, 38000 Grenoble, France; 30 Department of Infectious Diseases, University Hospital of Nancy, 54000 Nancy, France; 31 Médecine Intensive—Réanimation, Centre Hospitalier Régional d’Orléans, 45000 Orléans, France; 32 Département de Pharmacie, Centre Hospitalier Sud Francilien, 91100 Corbeil-Essonnes, France; 33 Critical Care Center, Ajaccio Hospital, 20000 Ajaccio, France; 34 Department of Clinical Pharmacy, Cochin Hospital, AP-HP, 75014 Paris, France; 35 Institut Cochin, INSERM U1016, CNRS UMR 8104, 75014 Paris, France; 36 INSERM, EFS BFC, UMR 1098 RIGHT, University of Bourgogne Franche-Comté, 25056 Besançon, France

**Keywords:** remdesivir, COVID-19, survival, pneumonia, SARS-CoV-2

## Abstract

Background. Given the rapidly evolving pandemic of COVID-19 in 2020, authorities focused on the repurposing of available drugs to develop timely and cost-effective therapeutic strategies. Evidence suggested the potential utility of remdesivir in the framework of an early access program. REMDECO-19 is a multicenter national cohort study assessing the ability of remdesivir to improve the outcome of patients hospitalized with COVID-19. Methods. We conducted a retrospective real-life study that included all patients from the early access program of remdesivir in France. The primary endpoint was the clinical course evolution of critically ill and hospitalized COVID-19 patients treated with remdesivir. Secondary endpoints were the SOFA score evolution within 29 days following the admission and mortality at 29 and 90 days. Results. Eighty-five patients were enrolled in 22 sites from January to April 2020. The median WHO and SOFA scores were respectively reduced by two and six points between days 1 and 29. Improvement in the WHO-CPS and the SOFA score were observed in 83.5% and 79.3% of patients, respectively, from day 10. However, there was no effect of remdesivir on the 90-day survival based on the control cohort for hospitalized COVID-19 patients with invasive ventilation. Conclusions. SOFA score appeared to be an attractive approach to assess remdesivir efficacy and stratify its utilization or not in critically ill patients with COVID-19. This study brings a new clinical benchmark for therapeutic decision making and supports the use of remdesivir for some hospitalized COVID-19 patients.

## 1. Introduction

Coronavirus disease 2019 (COVID-19) is caused by the severe acute respiratory syndrome coronavirus 2 (SARS-CoV-2) inducing a significant morbidity and mortality rate [1]. In France, during the first pandemic wave of COVID-19, most patients were asymptomatic or had mild illness; however, 87,809 people (134 per 100,000) were hospitalized with COVID-19 and 15,661 people (24 per 100,000) died in hospital [2]. Risks factors associated with development of acute respiratory distress syndrome and death included older age, male sex, neutrophilia, organ dysfunction, coagulopathy, and elevated D-dimer levels [3]. Given the rapidly evolving COVID-19 pandemic during the first wave, health authorities focused on the repurposing of available drugs to develop timely and cost-effective therapeutic strategies [4]. To date, supportive care for hospitalized patients with COVID-19 including oxygen supply and the use of dexamethasone in patients who received invasive mechanical ventilation are still the cornerstone of the medical care [5]. Expanded access programs (EAP) were developed for various drugs and emergency use authorization (EUA). COVID-19 management has been greatly improved thanks to EAP that have permitted drug repositioning such as for remdesivir, baricitinib, tocilizumab, nirmatrelvir–ritonavir, and molnupiravir and early access to COVID-19 convalescent plasma, casirivimab–imdevimab, bamlanivimab–etesevimab, sotrovimab, tixagevimab-cilgavimab, and nirmatrelvir [6].

Remdesivir is a prodrug of a nucleotide analogue that is intracellularly metabolized to an analogue of adenosine triphosphate that inhibits viral RNA-dependent RNA polymerases. It has demonstrated broad-spectrum activity against members of several families, including filoviruses, paramyxoviruses, and coronaviruses [7,8,9] and has shown prophylactic and therapeutic efficacy in nonclinical models of these coronaviruses. Remdesivir was touted as a potential candidate drug for the treatment of COVID-19 [10]. EAP evaluated the safety and efficacy of remdesivir in patients with moderate or severe COVID-19 during 5 to 10 days (200 mg loading dose on day 1, followed by 100 mg daily for up to 10 days) [10,11,12,13]. ACTT-1, a randomized, double-blind clinical trial, showed that remdesivir was superior to placebo in shortening the time to recovery in adult patients with severe COVID-19 [14]. Subsequently, a randomized, open-label trial in patients with severe COVID-19 not requiring mechanical ventilation, showed no significant difference between a 5-day and 10-day course of remdesivir [15]. These results prompted the US Food and Drug Administration (FDA) to grant EUA on 1 May 2020 [16]. On the 22nd of October 2020, remdesivir became the first United States FDA-approved drug for the treatment of hospitalized COVID-19 patients [17]. DisCoVeRy, a randomized, controlled, open-label clinical trial, did not observed clinical benefit of remdesivir in hospitalized patients versus standard of care by the WHO seven-point ordinal scale [18]. Recently, the PINETREE study showed very promising results: among non-hospitalized patients at high risk for COVID-19 progression, a 3-day course of remdesivir had an acceptable safety profile and resulted in an 87% lower risk of hospitalization or death than placebo [19].

Facing mixed outcomes [20,21], we set up a real-life study assessing the efficacy of remdesivir to improve clinical features in critically ill patients hospitalized with COVID-19 and severe pneumonia.

## 2. Materials and Methods

### 2.1. Settings

This national cohort study analyzes all patients included in EAP of remdesivir in France during the first wave of COVID-19. A nominative access of remdesivir was authorized by the National Agency for the Safety of Medicines and Health Products (ANSM) and a pharmaceutical company (Gilead Sciences, Foster City, CA, USA). Approval of requests was reserved for patients meeting the following criteria: (1) adult hospitalized for COVID-19 with a positive SARS-CoV-2 RT-PCR and/or typical chest computed tomographic [CT] characterized by scan bilateral and peripheral predominant ground glass opacities not fully explained by effusions, lobar or lung collapse, or nodules, (2) an oxygen saturation of 94% or less, (3) a creatinine clearance above 30 mL per minute and serum levels of alanine aminotransferase (ALT) and aspartate aminotransferase (AST) less than five times the upper limit of the normal range, and (4) not included in an ongoing clinical trial involving COVID-19. For each approved case, French hospitals were supplied with a stock of remdesivir needed for a 10-day treatment, consisting of a loading dose of 200 mg intravenously on day 1, plus 100 mg daily for the following 9 days. Any concomitant supportive therapy was permitted. The exclusion criteria were patients not receiving at least one dose of remdesivir and patients already enrolled in a clinical trial investigating remdesivir. The study protocol was approved by the institutional review board (IRB-00011928). Informed consent was obtained from all individual participants included in the study. Data collection has been declared to the National Commission for Data Processing and Freedoms. This trial is registered with ClinicalTrials.gov, NCT04365725.

### 2.2. Outcomes

The primary outcome was the clinical course evolution of patients under treatment with remdesivir using a WHO clinical progression scale (WHO-CPS) [22]. The seven-point scale consisted of the following categories: 1, not hospitalized, no limitation of activities; 2, not hospitalized, limitation of activities; 3, hospitalized, not requiring supplemental oxygen; 4, hospitalized, requiring supplemental oxygen; 5, hospitalized, requiring nasal high-flow oxygen therapy or noninvasive mechanical ventilation; 6, hospitalized, requiring invasive mechanical ventilation or extracorporeal membrane oxygenation (ECMO); and 7, death. Prespecified secondary outcomes were the Sequential Organ Failure Assessment (SOFA) score evolution [23], duration without mechanical ventilation within 29 days of initiation of treatment with remdesivir, and mortality at 29 and 90 days after initiation of treatment with remdesivir. Data were collected daily from the day before remdesivir introduction to 90 days after or until discharge or death. Safety outcomes were adverse events as measured by investigators using the NCI Common Terminology Criteria for Adverse Events version 3.0 and graded on a 0 to 4 scale (0, normal; 4, life-threatening).

### 2.3. Statistical Analysis

Summary statistics, namely median with interquartile range (IQR), and percentages, are reported unless otherwise specified. Comparison of baseline characteristics across groups used the nonparametric Wilcoxon rank sum test or the exact Fisher test. To detail the temporal dynamics of illness severity of those patients, the evolution of patient trajectories across the WHO scale over time was illustrated using multistate models based on the time-inhomogeneous Markov chain [24].

The 90-day survival was compared between patients requiring invasive mechanical ventilation with a previously described cohort [25] meeting the same four criteria mentioned above and not receiving remdesivir. To consider a confounding bias related to the non-random choice of treatment, different weightings were used to correct the sample. These weightings estimate the average effects of the treatment on different populations: ATE (average treatment effect) on the population corresponding to the sample [26], ATT (average effect in treated), or ATO (on the population balanced in prognostic terms) [27,28]. We used the weightings allowing a balance on the prognostic factors, measured on the standardized mean differences (SMD). Any value >0.10 in the pooled standard deviation (SD) was considered unbalanced.

All analyses were performed on R 3.6.2 (https://www.R-project.org, accessed on 15 June 2022). The R package mstate was used to estimate the transition and state occupation probabilities. The PSweight and survey packages were used to compare the 90-day survivals. The study was designed and conducted by the sponsor Assistance Publique—Hôpitaux de Paris (AP-HP), in accordance with the protocol. The sponsor collected the data, monitored conduct of the program, and performed the statistical analyses.

## 3. Results

### 3.1. Characteristics of Study Participants and Treatment

Eighty-five patients were enrolled in 22 sites from 24 January 2020 to 21 April 2020. The median age at inclusion was 60 years (interquartile range, IQR, 49 to 69), ranging from 25 to 85 years (Table 1). Sixty-six patients (77.6%) were men. At baseline, most patients (n = 71; 83.5%) received invasive ventilation in the intensive care unit (ICU); thirteen (15.3%) were receiving non-invasive ventilation in the intermediate care unit; and only one patient (1.2%) did not require oxygen in conventional hospitalization. Before initiating treatment with remdesivir, the median duration of symptoms was 11 days (IQR, 8 to 14) and the median duration of COVID-19 diagnosis was 5 days (IQR, 2 to 7). Those data did not differ substantially between patients receiving invasive ventilation and those receiving non-invasive ventilation. Patients had invasive mechanical ventilation for 2 days (range, 1–6) before the initiation of remdesivir. Patients who were receiving non-invasive oxygen support at baseline were younger (median age, 59 years, vs. 63 years) than those receiving invasive ventilation and were more likely to be female (22.5%, vs. 15.4%) and had higher prevalence of diabetes (14.1%, vs. 7.7%) and respiratory pathology (19.7%, vs. 15.4%). Median body mass index (BMI) and median laboratory values were similar between these groups. At baseline, patient laboratory analyses were characterized by elevated liver markers (ASAT and ALAT > 5 N, LDH), normal renal function, and normal white blood cell count despite a lymphopenia. Median WHO and SOFA scores at remdesivir initiation were six and seven, respectively.

All patients received at least one dose of remdesivir from 29 January 2020 to 24 April 2020. Fifty-five patients (64.7%) received the full 10-day course of remdesivir, 22 patients (25.9%) received five to nine days of treatment, and eight patients (9.4%) received remdesivir for less than five days. Remdesivir discontinuation before 10 days was caused by limiting toxicity or contraindication (n = 12; 14.1%), clinical improvement (n = 8; 9.4%), therapeutic strategy change (n = 6; 7.1%), or death (n = 5; 5.9%). Forty-four patients (51.8%) received concomitant COVID-19 treatments: 22 (25.9%) received in addition hydroxychloroquine, 23 (27.1%) received lopinavir/ritonavir, 11 (12.9%) received corticoids, 6 (7.1%) received azithromycin, and 3 (3.6%) received other treatments. At baseline, 32 patients (37.6%) received vasopressor treatment, of whom 31 were under invasive ventilation and 1 had non-invasive oxygen support.

### 3.2. Control Group

**Figure 1:**
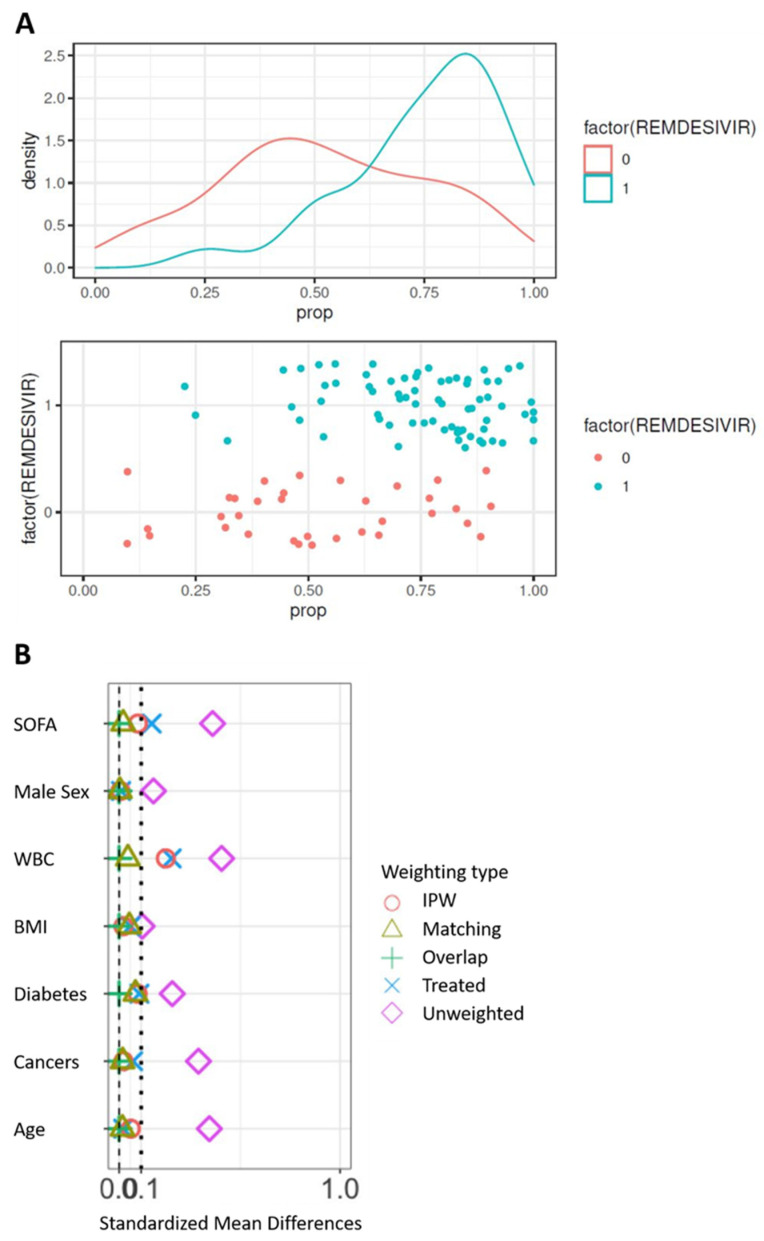
Correction of confusion bias by weighting. (**A**) Distribution of the propensity score between groups on the original basis. (**B**) Correction of the imbalances according to the types of weighting. WBC, white blood cells; BMI, body mass index.

The control cohort for the comparison of 90-day survival is presented in Supplemental Table S1. The propensity score was constructed from the seven following variables: age, sex, BMI, SOFA score at admission, white blood cells at admission, and comorbidities (diabetes and cancer). At first, the model was built on complete cases, i.e., in 105 patients on invasive ventilation, including 71 patients from the remdesivir group. Significant differences are observed between groups (Table 2) with SMD > 0.10 on the seven prognostic factors. In particular, the subjects treated were younger and had a lower SOFA than the controls before the correction of the confusion bias. Difference in both treatment groups can be displayed by the distribution of the propensity score. The propensity score to have received remdesivir was estimated using a multivariate logistic model including the seven unbalanced prognostic factors. The distribution of the score in the two groups on the original basis (Figure 1A) validates the hypothesis of common support. Weightings were applied from the propensity score, resulting in pseudo-populations. The correction of the imbalances was evaluated on the bases thus weighted (Figure 1B). Only weightings with matching or overlapped weights allow all SMDs to be reduced below 0.10. These two populations were those subsequently retained.

### 3.3. Mortality

Twelve out of 85 patients (14.1%) died before the 29 days of follow-up was completed, all with invasive ventilation at baseline. The median age of these patients was 76.5 years (IQR, 66 to 80). The median time between remdesivir initiation and death was 16 days.

Regarding the patients under invasive ventilation, 12 (16.9%) in the remdesivir group died within 90 days after the start of hospitalization, compared with 12 (35.29%) in the control arm (Table 3).

### 3.4. WHO Score

**Figure 2:**
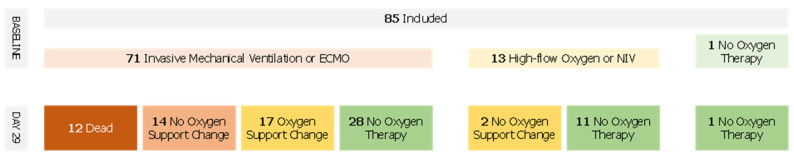
Oxygen Support Flow Chart.

**Figure 3:**
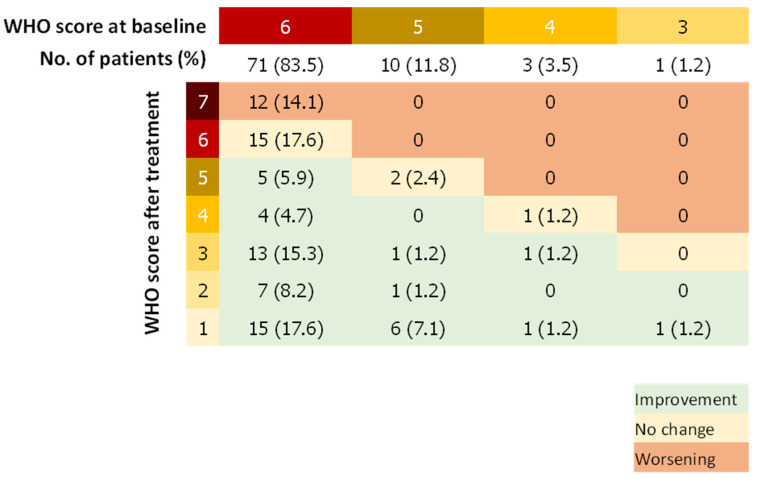
WHO Score Double-Entry Board. WHO score at baseline and after remdesivir treatment; Number (%).

The median WHO score was reduced by two points (IQR, 0 to 4) between days 1 and 29. Forty-eight patients (56.5%) showed a WHO score reduced by at least 2 points whereas 14 patients (16.5%) had a worsening WHO score during the follow-up (Figure 2). Fifty-seven patients (67.9%) improved the category of oxygen support over a median of 9 days after the first dose of remdesivir. Median duration to change category of oxygen support was 12.5 (IQR, 8.8–17) and 14 (IQR, 8 to 18) days for patients treated with remdesivir within 11 days of symptoms onset and more than 11 days after symptoms onset, respectively. Among patients on invasive ventilation at baseline, (n = 71, 83.5%), 27 (31.7%) did not improve their WHO score after remdesivir treatment, of which 12 died (14.1%), whereas 28 patients (39.4%) were extubated or stopped receiving ECMO after the 29 days of follow-up. Among patients not on invasive ventilation (n = 13, 16.5%), 11 (84.6%) stopped receiving any oxygen and none worsened according to the WHO scale (Figure 3).

### 3.5. SOFA Score

**Figure 4:**
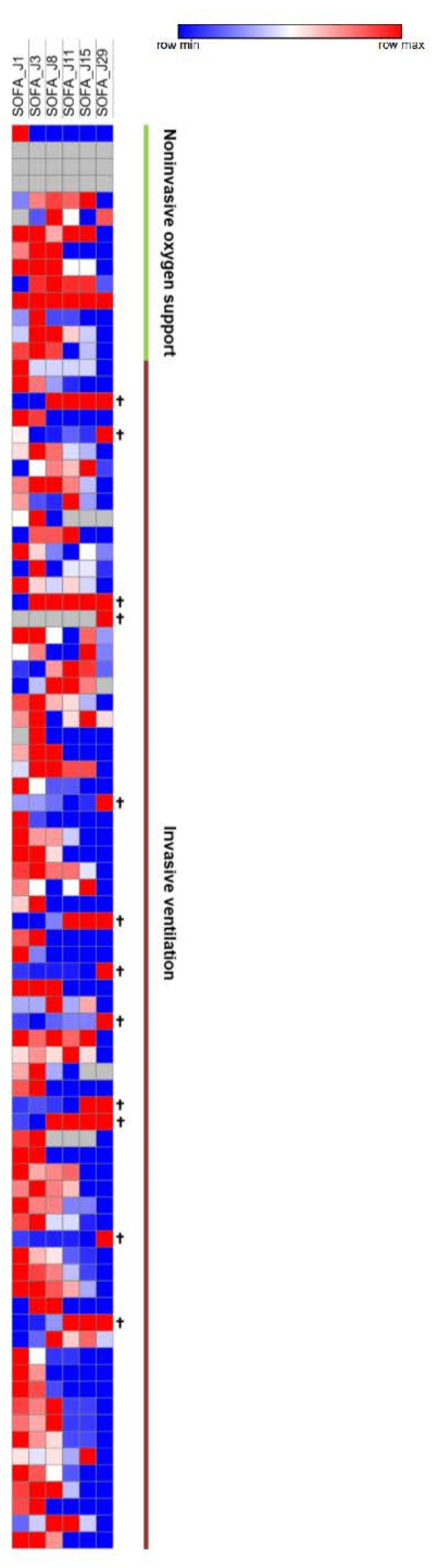
SOFA Scores Heatmap. SOFA scores at day 1, 3, 8, 11, 15, and 29. Increasingly blue colors mean improved SOFA scores compared to J1; increasingly red colors mean worsened SOFA scores compared to J1. Each row corresponds to a patient.

**Figure 5:**
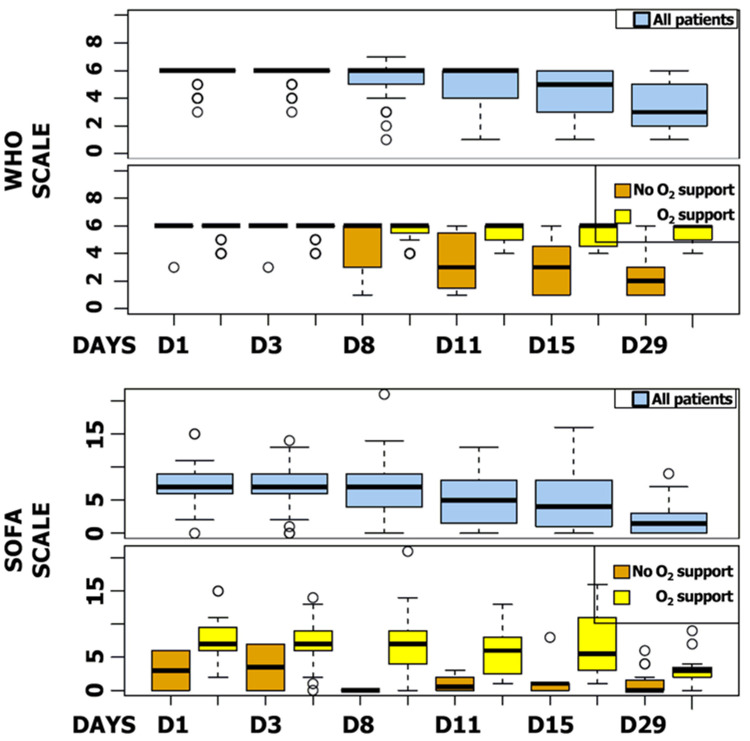
SOFA and WHO Scores Box Plot. SOFA and WHO scores at day 1, 3, 8, 11, 15, and 29 for all patients and regarding oxygen support.

The median SOFA score was reduced by six points during follow-up (IQR, 2 to 8). Five patients (5.9%) showed a worsening SOFA score, including three patients receiving a concomitant COVID-19 treatment. The majority of patients improved their SOFA score at day 11 (n = 65, 79.3%), particularly patients receiving invasive ventilation at baseline (87.3%) (Figure 4). WHO and SOFA scores evolution depending or not on oxygen requirement are represented in Figure 5. The median duration of vasopressor treatments was 8.5 days and the median duration between the first day of remdesivir treatment and the end of the vasopressive treatment was 6 days. At the end of follow-up, 49 patients (57.6%) were still hospitalized and 22 were still oxygen-requiring including 14 still under invasive ventilation.

### 3.6. Safety

The most common adverse events were increased hepatic enzymes (31.8%), anemia (30.6%), renal impairment (28.2%), and hypotension (23.5%) (Table 4). Adverse events occurred both in patients with invasive and non-invasive ventilations (70.4% vs. 69.2%). Thirty-eight patients (44.7%) had serious adverse events. The most common serious adverse events were hypotension (22.3%), anemia (14.1%), renal impairment (15.3%), and deep-vein thrombosis (12.9%). Eleven patients (12.9%) discontinued remdesivir treatment prematurely because of limiting toxicity.

## 4. Discussion

This retrospective, national multicenter study investigated the clinical evolution in real life of all patients with severe COVID-19 pneumonia treated in France with remdesivir outside of clinical trials during the first pandemic wave of COVID-19. Our cohort included a majority of patients in the most severe COVID-19 forms, namely requiring invasive mechanical ventilation and high-flow oxygen, and a majority of men. This patient description at admission is consistent with previous single-arm studies on remdesivir treatment for severe COVID-19 [10,16]. We showed an improvement in oxygen-support status for 67.9% of patients and an overall mortality of 14.1% over a 29-day follow-up. These clinical outcomes were consistent with the literature. Grein’s et al. described a 68% improvement in oxygen-support status [10]. Randomized, controlled trials reported an all-cause mortality rate of 11.4% [14] or 14% [29]. Improvement in the ordinal scale score was observed in 83.5% of cases and probability of change in WHO score over time was higher between 10 to 15 days following remdesivir introduction. At the same time, 79.3% of patients showed an improvement for the SOFA score from day 11 of the follow-up. At the end of the 29 days of follow-up, 36 patients (42.4%) were discharged from hospital.

In terms of safety, real-life observations are essential in addition to clinical trials—all the more so since the results of the trials are difficult to transpose to the general population, the patients most at risk of adverse effects being less often included. Mild-to-moderate elevations in ALAT, ASAT, or both were observed in this cohort, as reported [30,31]. Renal abnormalities—elevations in creatinine and declines in creatinine clearance—were also observed. However, COVID-19 itself has been found to be associated with liver and renal injuries [32,33,34]. Thus, attribution of hepatotoxicity or renal toxicity to either remdesivir or the underlying disease is challenging without control cohorts.

In addition to the efficacy results generally reported through a WHO scale dedicated to COVID-19, our study has the advantage of analyzing the SOFA, a score usually used in ICU and reflecting the clinical evolution of this category of critical patients. To our knowledge, no other study has looked at SOFA score as an efficacy feature of remdesivir. Widely established to monitor the patient’s condition in an ICU, this score was used to determine organ function or failure rate over time [23,35] so it might be an attractive approach to assess remdesivir efficacy in standard care. It included components reflective of the respiratory, coagulation, liver, cardiovascular, renal, and central nervous systems. Mine et al., had demonstrated that models based on SOFA scores at admission had performance in predicting mortality in patients in ICUs [36]. Some studies focused on this score during the COVID-19 pandemic to assess patient outcome following mechanical ventilation [37,38]. As a result, a decreased SOFA score over time in mechanically ventilated patients is associated with ICU survival. Moreover, the association between decreased SOFA score over time and survival was independent of comorbidities [38]. SOFA score could function as an effective adjunctive risk stratification tool at admission for critical COVID-19 patients [37] and allowed the identification of patients at risk of further deterioration and mortality. In our study, 65 out of the 85 patients showed an improvement of SOFA score from the 11th day of the follow-up. Although previous studies report on SOFA score in COVID-19, data are still sparse and disparate. Admission SOFA score seems not to be associated with ICU death and some individual components such as respiratory, circulatory, or renal functions of the SOFA score are more critical [38]. In our study, 19 patients had a SOFA score greater than 10 at baseline. Among them, 26% died versus 10% among those with a baseline SOFA score below 10.

The comparison of our results with a control cohort using a propensity score method to balance the cohorts, shows that treatment with remdesivir does not improve survival at D90 for patients under invasive ventilation. Based on the matched and the overlapped samples, there was no effect of remdesivir on the 90-day survival. Our real-life study confirms the literature data. No randomized trial has demonstrated an improvement for remdesivir on mortality in hospitalized COVID-19 patients. In mild to moderate forms of COVID-19, despite the results of the PINETREE trial, remdesivir no longer seems to have a place in the therapeutic arsenal in view of the recent approvals of nirmatrelvir–ritonavir and sotrovimab in this indication [37,38]. Finally, a well-known challenge in these studies is potential missing data. Despite these limitations, real-life study of remdesivir use in patients with severe COVID-19 pneumonia is a major issue to confirm the results of controlled clinical trials with data from routine care, thus reflecting current practice.

## 5. Conclusions

SOFA score appeared to be an attractive approach to assess remdesivir efficacy and stratify its utilization or not in critically ill patients with COVID-19. This study brings a new clinical benchmark for therapeutic decision making and supports the use of remdesivir for some hospitalized COVID-19 patients with a SOFA score below 10 but this remains to be demonstrated in a randomized controlled trial.

## Figures and Tables

**Table 1:** Baseline Demographic and Clinical Characteristics of the Patients.

	Invasive Ventilation (n = 71)	Noninvasive Oxygen Support (n = 14)	Total (n = 85)
Characteristics			
Median age (IQR)—years	59 (48.5–69)	63 (54–71)	60 (49–69)
Age category—no. (%)			
<50	20 (28.2)	2 (15.4)	23 (27.1)
50 to <70	35 (49.3)	6 (46.2)	41 (48.2)
>70	16 (22.5)	5 (38.5)	21 (24.7)
Male sex—no. (%)	55 (77.5)	11 (84.6)	66 (77.6)
Oxygen-support category—no. (%)			
Invasive ventilation	71 (100)	0	71 (83.5)
Non-invasive oxygen support		13 (100)	13 (15.3)
Of which high-flow oxygen		7 (53.8)	7 (8.2)
Median duration of symptoms before remdesivir therapy (IQR)—days	11 (9–14)	9 (6–12)	11 (8–14)
Median duration of symptoms before admission (IQR)—days	6 (4–8)	5 (5–7)	6 (4–8)
Median duration of diagnosis before remdesivir therapy (IQR)—days	5 (2–7)	3 (1–4)	5 (2–7)
Coexisting conditions—no. (%)			
Any condition	50 (70.4)	9 (69.2)	60 (70.6)
Cardiovascular	35 (49.3)	8 (61.5)	44 (73.3)
Diabetes	10 (14.1)	1 (7.7)	11 (18.3)
Dyslipidemia	12 (16.9)	5 (38.5)	17 (28.3)
Respiratory pathology	14 (19.7)	2 (15.4)	16 (26.7)
Cancer	3 (4.2)	0	3 (5)
Median BMI (IQR)	27.8 (26.4–31.4)	27.7 (25.6–33.2)	27.8 (26.2–31.4)
Normal no. (%)	11 (15.5)	2 (15.4)	14 (18.0)
Overweight no. (%)	33 (46.5)	4 (30.8)	37 (47.4)
Moderate obesity no. (%)	16 (22.5)	3 (23.1)	19 (24.4)
Severe obesity no. (%)	4 (5.6)	0	4 (5.1)
Morbid obesity no. (%)	3 (4.2)	1 (7.8)	4 (5.1)
Median WHO score (Min—Max)	6 (6–6)	5 (4–5)	6 (3–6)
Median SOFA score (Min—Max)	8 (1–15)	3 (2–7)	7 (0–15)
Median laboratory values (IQR)			
ASAT—IU per liter	49 (37–68)	42 (30–77)	49 (37–68)
ALAT—IU per liter	40 (27–60.3)	45 (32.5–72)	40.5 (27–64)
LDH—IU per liter	385 (343.5–469.8)	587 (375–631)	387 (344–503)
Creatinine—µmol per liter	71.5 (55.8–89.3)	74 (58.5–85.5)	72 (56.8–89)
Bilirubin—µmol per liter	9.8 (7.7–16.9)	8 (6–12)	9.1 (7.5–16)
Hemoglobin—g per deciliter	12.1 (10–13)	13.5 (12.1–14.5)	12.3 (10.3–13.4)
Platelets—Giga/L	162 (0.384–259)	174 (0.252–217.5)	163 (0.359–252)
WBC—Giga/L	8.7 (6.655–12.395)	6.36 (4.95–8.275)	8.465 (6.125–11.96)
Lymphocytes—Giga/L	0.90 (0.572–1.64)	0.87 (0.555–1.125)	0.9 (0.55–1.48)

ALAT alanine aminotransferase, ASAT aspartate aminotransferase, BMI body mass index, IQR interquartile range, LDH lactate dehydrogenase, SOFA sequential organ failure assessment, WBC white blood cell, WHO world health organization.

**Table 2:** Comparison of patients on invasive ventilation with controls.

	Control (n = 34)	Remdesivir (n = 71)	SMD	*p*-Value
Characteristics				
Age—mean (SD)	63.4 (12.93)	57.8 (14.22)	0.408	0.054
Male sex—% (SD)	0.71 (0.46)	0.77 (0.42)	0.156	0.47
BMI—mean (SD)	28.4 (7.15)	29.04 (4.67)	0.104	0.24
SOFA J0—mean (SD)	9.59 (3.57)	8.18 (3.05)	0.423	0.040
WBC—mean (SD)	7608.82 (3395.48)	13,269.72 (16,920.34)	0.464	0.001
Diabetes—mean (SD)	0.24 (0.43)	0.14 (0.35)	0.241	0.27
Cancer—mean (SD)	0.15 (0.36)	0.04 (0.20)	0.359	0.11

**Table 3:** Comparison of 90-day survival between remdesivir and control cohorts.

	Estimate	Standard Error	T Value	Pr (>|t|)
Unweighted	0.18	0.09	2.13	0.04
Matching	0.14	0.10	1.37	0.17
Overlap	0.16	0.10	1.58	0.11

**Table 4:** Adverse events.

	Total (n = 85)
**Event**	
**Any adverse event**	**59 (69.4)**
Hepatic enzyme increased	27 (31.8)
Anemia	26 (30.6)
Thrombopenia	9 (10.6)
Neutropenia	4 (4.7)
Diarrhea	10 (11.8)
Rash	6 (7.1)
Acute kidney injury	24 (28.2)
Hypotension	20 (23.5)
Atrial fibrillation	9 (10.6)
Multiple organ dysfunction syndrome	5 (5.9)
Hypernatremia	6 (7.1)
Deep-vein thrombosis	14 (16.5)
Acute respiratory distress syndrome	19 (22.3)
Pneumothorax	5 (5.9)
Hematuria	10 (11.8)
Delirium	6 (7.1)
Septic shock	10 (11.8)
**Any serious adverse event**	**38 (44.7)**
Hepatic enzyme increased	9 (10.6)
Anemia	12 (14.1)
Thrombopenia	3 (3.5)
Neutropenia	4 (4.7)
Diarrhea	4 (4.7)
Rash	4 (4.7)
Acute kidney injury	13 (15.3)
Hypotension	19 (22.3)
Atrial fibrillation	9 (10.6)
Hypernatremia	3 (3.5)
Deep-vein thrombosis	11 (12.9)
Pneumothorax	4 (4.7)
Hematuria	5 (5.9)
Delirium	5 (5.9)

## Data Availability

The datasets generated during and/or analyzed during the current study are available from the corresponding author on reasonable request.

## Supplementary Materials

The following supporting information can be downloaded at: https://www.mdpi.com/article/10.3390/jcm11216545/s1, Table S1: Control and remdesivir cohorts.

## Abbreviations

BMI	Body mass index
COVID-19	Coronavirus disease 2019
SARS-CoV-2	Severe acute respiratory syndrome coronavirus 2
EAP	Expanded access programs
EUA	Emergency use authorization
FDA	Food and Drug Administration
ANSM	National Agency for the Safety of Medicines and Health Products
CT	Computed tomographic
ALT	Alanine aminotransferase
AST	Aspartate aminotransferase
IRB	Institutional review board
WHO-CPS	World Health Organization Clinical Progression Scale
SOFA	Sequential Organ Failure Assessment
ECMO	Extracorporeal membrane oxygenation
IQR	Interquartile range
ATE	Average treatment effect
ATT	Average treatment effect in treated
ATO	Average treatment effect in the overlap
SMD	Standardized mean differences
SD	Standard deviation
AP-HP	Assistance Publique—Hôpitaux de Paris
ICU	Intensive care unit
