# Supplementary material for: Remdesivir for Patients Hospitalized with COVID-19 Severe Pneumonia: A National Cohort Study (Remdeco-19)
Source: J Clin Med. 2022 Nov 4;11(21):6545. doi: 10.3390/jcm11216545 (PMC9654065; doi:10.3390/jcm11216545)
Supplement: Supplementary file 1 [file jcm-11-06545-s001.zip › jcm-1939600-supplementary.pdf]

ADDITIONAL FILE

Supplementary Table S1. Control and remdesivir cohorts.

REMDESIVIR	Age	SexH=1	BMI	SOFA Day 0	Leukocytes Day 0	Diabete	Cancer	Alive Day 90
0	42	1	32	3	5800	0	0	0
0	70	0	25	11	7900	0	0	1
0	81	1	27	9	3100	0	1	0
0	70	0	29	5	11200	0	0	0
0	78	1	26	5	9700	1	0	0
0	55	0	32	4	5900	0	1	1
0	54	1	26	9	3500	1	0	1
0	63	1	22	12	6900	0	0	1
0	80	1	24	9	4000	0	0	1
0	77	1	24	6	6800	0	1	0
0	76	1	29	6	6400	1	0	0
0	79	0	19	4	10000	0	0	1
0	55	0	35	10	2500	0	0	1
0	58	1	26	11	3000	0	0	1
0	40	1	23	7	4500	1	0	1
0	75	1	26	14	5500	1	0	0
0	74	0	30	9	2600	0	1	0
0	83	0	20	14	5100	0	0	1
0	76	1	26	10	8000	1	0	0
0	74	1	33	5	4900	0	1	0
0	52	1	30	10	7000	0	0	1
0	69	1	30	4	7200	0	0	1
0	63	1	27	13	8500	0	0	1
0	61	0	63	14	9000	1	0	1
0	61	0	32	15	9000	0	0	1
0	57	1	29	12	10800	1	0	0
0	45	1	26	13	13400	0	0	1
0	50	1	29	11	7100	0	0	1
0	61	1	29	14	6900	0	0	1
0	62	1	28	8	14400	0	0	1
0	38	0	23	13	10000	0	0	1
0	42	1	33	14	15900	0	0	0
0	69	1	26	11	9800	0	0	1

0	64	1	27	11	12400	0	0	1
1	61	1	25	13	8700	0	0	1
1	50	0	28	13	8400	0	0	1
1	77	1	27	13	17700	0	0	0
1	65	1	25	9	36630	0	1	1
1	60	1	28	14	11200	0	0	0
1	40	0	27	8	5000	1	1	1
1	37	1	27	3	8340	0	0	1
1	73	1	34	13	8500	1	0	1
1	51	1	27	9	7280	0	0	1
1	52	1	27	10	10210	1	0	1
1	67	0	25	8	11010	0	1	1
1	59	0	36	13	8430	0	0	1
1	54	0	36	2	8540	1	0	1
1	43	1	21	14	12910	0	0	1
1	70	1	29	15	11500	0	0	0
1	80	1	29	8	12500	0	0	0
1	42	1	26	11	6600	0	0	1
1	39	1	28	9	11200	0	0	1
1	35	0	36	6	45500	0	0	1
1	49	1	31	3	15990	0	0	1
1	25	1	26	12	15740	0	0	1
1	38	1	40	9	5120	0	0	1
1	61	1	29	9	12000	0	0	1
1	40	1	27	6	6000	0	0	1
1	69	0	32	5	6000	1	0	1
1	71	1	31	6	6040	0	0	1
1	76	0	27	8	13620	0	0	0
1	41	1	32	7	15990	0	0	1
1	70	1	25	10	11240	0	0	1
1	80	0	28	7	11100	0	0	1
1	63	1	23	8	78000	0	0	1
1	76	1	30	10	6910	0	0	1
1	58	1	32	6	6710	0	0	1
1	68	1	28	8	12290	0	0	0
1	67	1	32	5	5000	0	0	1
1	70	0	25	4	9000	0	0	1
1	60	0	46	6	13000	0	0	0
1	52	1	33	3	7000	0	0	1
1	49	1	28	4	6000	0	0	1
1	81	1	29	10	12000	0	0	0
1	61	1	44	8	7500	0	0	1
1	57	0	38	7	10600	0	0	1
1	69	1	30	10	9900	0	0	1

1	40	0	29	5	4400	1	0	1
1	85	0	27	7	13100	0	0	0
1	80	1	23	11	14200	0	0	0
1	53	1	22	8	12500	0	0	1
1	74	1	34	7	17800	1	0	1
1	75	1	22	15	7000	0	0	1
1	69	1	28	8	8700	0	0	1
1	57	1	28	8	11450	0	0	1
1	62	1	29	12	6100	0	0	1
1	79	1	29	9	12600	1	0	0
1	60	1	31	11	15700	0	0	1
1	46	1	29	8	6200	0	0	1
1	65	1	29	8	126000	0	0	1
1	52	1	23	5	8050	1	0	1
1	54	0	31	7	10600	0	0	0
1	54	1	33	7	10000	0	0	1
1	43	1	23	10	3000	0	0	1
1	43	1	27	7	12000	0	0	1
1	29	1	26	7	8400	0	0	1
1	66	1	24	7	8100	0	0	1
1	35	1	27	7	7200	0	0	1
1	48	1	25	7	11460	0	0	1
1	55	1	32	7	11830	0	0	1
1	50	1	30	7	8590	0	0	1
1	68	0	26	10	8400	0	0	1
1	50	1	28	6	6800	0	0	1
1	67	1	31	1	6300	0	0	1
1	39	1	29	7	12770	1	0	1
